# *Francisella tularensis* Subspecies *holarctica*, Tasmania, Australia, 2011

**DOI:** 10.3201/eid1809.111856

**Published:** 2012-09

**Authors:** Justin Jackson, Alistair McGregor, Louise Cooley, Jimmy Ng, Mitchell Brown, Chong Wei Ong, Catharine Darcy, Vitali Sintchenko

**Affiliations:** Royal Hobart Hospital, Hobart, Tasmania, Australia (J. Jackson, A.R. McGregor, L. Cooley, C.W. Ong, C. Darcy);; Westmead Hospital, Sydney, New South Wales, Australia (J. Ng, M. Brown, V. Sintchenko);; and The University of Sydney, Sydney (V. Sintchenko)

**Keywords:** *Francisella tularensis* subtype *holarctica*, possum, opossum, marsupial, ulceroglandular, tularemia, Australia, molecular detection, bacteria, antimicrobial drugs

## Abstract

We report a case of ulceroglandular tularemia that developed in a woman after she was bitten by a ringtail possum (*Pseudocheirus peregrinus*) in a forest in Tasmania, Australia. *Francisella tularensis* subspecies *holarctica* was identified. This case indicates the emergence of *F. tularensis* type B in the Southern Hemisphere.

Tularemia is a zoonosis affecting a wide range of wildlife species, including mammals, birds, amphibians, and arthropods (*1*,*2*). Three subspecies of *Francisella tularensis* have been recognized as causes of disease in humans: ssp. *tularensis* (type A tularemia), ssp. *holarctica* (type B tularemia), and ssp. *novicida*. Type B tularemia is endemic to the Northern Hemisphere, and cases predominately occur in latitudes 30°N–71°N (*1*,*2*). We report a case of ulceroglandular tularemia in a human in Tasmania, Australia (latitude 42°S) who was bitten by a ringtail possum (*Pseudocheirus peregrinus*, Figure 1).

**Figure 1:**
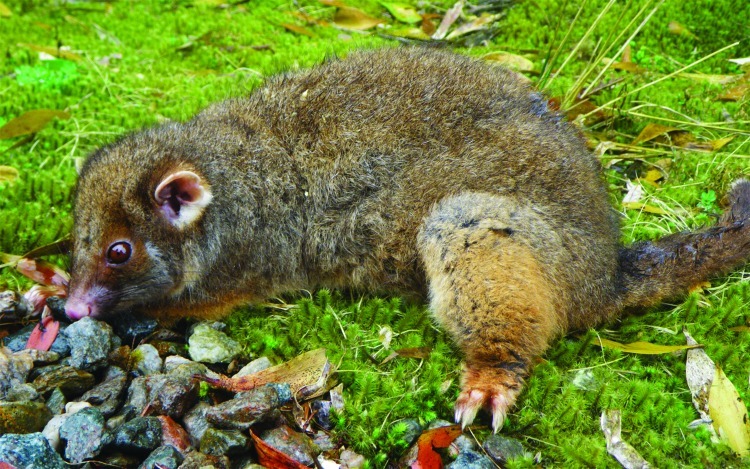
Ringtail possum (*Pseudocheirus peregrinus*) as photographed by its bite victim, Tasmania, Australia, 2011.

## The Case

In February 2011, a 44-year-old woman was bitten on her right index finger by a wild ringtail possum near the Henty River on the western coast of Tasmania. The incident occurred in daylight, and the possum, normally nocturnal, appeared lethargic and unwell. The patient was immunocompetent and had never traveled outside of Australia. Three days later, an ulcer developed at the site of the bite, followed by the development of swollen and tender epitrochlear lymph nodes, fever, rigors, myalgias, and drenching night sweats. On day 4, the patient was prescribed oral β-lactam antimicrobial drugs by her local doctor. She took these for 2 weeks without clinical improvement. Axillary lymphadenopathy was palpable on day 14, and by day 17 the epitrochlear nodes had formed a spontaneously discharging sinus. A swab sample was collected from the primary ulcer site on day 17. No organisms grew after a 48-h incubation at 36°C in the following culture media: blood agar in ambient air, MacConkey agar in ambient air, and chocolate agar in 5% CO_2_. The patient’s antimicrobial drug regimen was altered empirically to ciprofloxacin 500 mg 2×/day and amoxicillin/clavulanic acid 875 mg/125 mg 2×/day for the next 4 weeks. Despite mild improvement in symptoms, epitrochlear and axillary lymphadenitis with suppuration persisted. Swab samples collected for fungal and mycobacterial culture from the epitrochlear sinus on day 30 were culture negative. On day 47, the patient was referred to the Royal Hobart Hospital in Tasmania. Histology from an excisional biopsy of the epitrochlear sinus on day 50 revealed acute inflammation and a few non-necrotizing epithelioid granulomas. This tissue and an axillary lymph node aspirate were sent to a reference laboratory for molecular studies.

**Figure 2:**
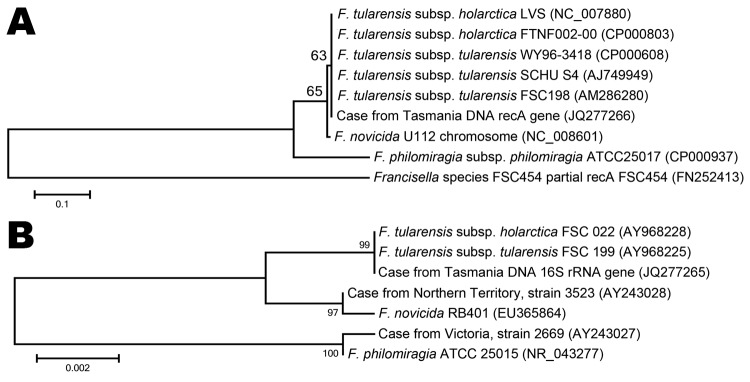
Nucleotide sequence comparison of the *recA* (A) and 16S rRNA (B) genes of *Francisella tularensis* subspecies *holarctica*, Tasmania, Australia, 2011. Reference sequences from the GenBank/European Molecular Biology Laboratory/DNA DataBank of Japan databases and 16S rRNA gene sequences from an *F. novicida* and an *F. philomiragia* infection reported from Australia (*4*) were aligned with amplicons of 16S rRNA and *recA* genes from samples with PCR results positive for *F. tularensis* from a 44-year-old woman. Scale bars indicate nucleotide substitutions per site.

In the reference laboratory, the 16S rRNA gene was amplified from the axillary aspirate by using primers targeting the U1, U3, and U5 regions. The resulting 1,331-bp amplicon (GenBank accession no. JQ277265) demonstrated 100% homology with 16S rRNA gene sequences from *F. tularensis* ssp. *tularensis* and *holarctica* stored in GenBank. PCR and sequencing of the *recA* gene (*3*) confirmed *F. tularensis* (GenBank accession no. JQ277266). Amplicons of 16S rRNA and *recA* genes were aligned with reference sequences from the GenBank/European Molecular Biology Laboratory/DNA DataBank of Japan databases and with sequences from 2 previous *Francisella* spp. reported from Australia (*4*). The 16S rRNA and *recA* gene sequences showed 100% query coverage and an Expect value of 0.00 in BLASTn (http://blast.ncbi.nlm.nih.gov/Blast.cgi) with 36 submissions of *F. tularensis* genomes (Figure 2). The unrooted bootstrap consensus tree generated from sequence alignment of the *recA* gene sequence, coupled with the neighbor-joining method, suggested homology between *F. tularensis* ssp. *holarctica* and the organism responsible for this patient’s illness. Sequencing of the region of difference 1 (*5*) by using previously validated primers (*3*) indicated the region of difference 1 size was 835 bp, closely approximating the 840-bp size associated with *F. tularensis* ssp. *holarctica* biovar *japonica*.

Lymph node aspirate from the patient cultured in horse blood and in blood cysteine and chocolate media with and without supplement (BBL IsoVitaleX Enrichment; Becton, Dickinson and Co., Sparks, MD, USA) produced no growth after 2 weeks of incubation (37°C, 5% CO_2_); the control strain of *F. tularensis* live vaccine strain grew within 3 days. We used reagents and protocols from the Laboratory Response Network, US Centers for Disease Control and Prevention, to perform real-time PCR for *F. tularensis*. The procedure amplified 2 of 3 targets (FT2 and FT3). IgG and IgM against *F. tularensis* antigens were detected by use of ELISA (Virion-Serion, Würzburg, Germany) in serum obtained 2 months after the onset of symptoms in concentrations of 147.13 and 222.74 U/mL, respectively (positive cutoff >15 U/mL). Confirmatory serology for *F. tularensis* was performed by the Centers for Disease Control and Prevention vector-borne diseases laboratory (Fort Collins, CO, USA) on serum samples collected 56 and 75 days after symptom onset. Both specimens were positive for *F. tularensis*, evidenced by a microagglutination titer of 128 (positive cutoff >128). On the basis of these results, the patient was treated with a 14-day course of intravenous gentamicin. Her symptoms resolved promptly, and the woman remained well at a 6-month follow-up visit.

This case demonstrates the well-recognized features of *F. tularensis* ssp. *holarctica* infection as described from the Northern Hemisphere. The mode of transmission, incubation period, clinical syndrome, lack of response to β-lactam antimicrobial drugs, and response to aminoglycoside therapy are all characteristic of type B tularemia (*6*). Although a clinical response to ciprofloxacin could have been expected, treatment failures have been reported when initiation of therapy is delayed (*7*). The lack of growth on blood and chocolate agar is also consistent with ssp. *holarctica* and contrasts with ssp. *novicida* and *F. philomiragia* (*4*,*6*). The inability to culture an organism on specialized media probably was caused by prolonged ciprofloxacin treatment before the specific culture attempt. This case met the definition for probable tularemia according to the 2007 World Health Organization guidelines (*8*); however, the combination of clinical, serologic, and molecular evidence strongly supports the diagnosis of infection with *F. tularensis* ssp. *holarctica*. This conclusion is consistent with recent reports of tularemia in which case definitions included a compatible clinical syndrome and positive *F. tularensis* real-time PCR or DNA sequencing (*9*,*10*).

Cases of human infection with other *Francisella* species have been reported from Australia (*4*). *F. philomiragia* was isolated from a lymph node in a child in Victoria with chronic granulomatous disease, and a *novicida*-like organism was cultured from the toe of a 53-year-old man after a cut received in brackish water in the Northern Territory. In contrast to type A and B tularemia, these organisms are rare human pathogens and are not associated with arthropod vectors or animal hosts.

More than 300 species of mammals, birds, invertebrates, and amphibians are currently recognized as being susceptible to type B tularemia (*1*). This report adds the ringtail possum, found widely throughout Australia, to the list of species that are susceptible to tularemia and have transmitted this pathogen to humans. Although transmission of tularemia is most often associated with species from the orders Lagomorpha and Rodentia, human infection following contact with marsupials has long been recognized. Within the United States, opossum-to-human transmission was described as early as 1929 (*11*).

The discovery of *F. tularensis* in this remote location of Australia raises questions about possible routes of spread and natural reservoirs of tularemia. Tularemia has emerged several times in nonendemic areas after the importation of infected wildlife (*12*) or changes in ecologic and climate conditions (*13*), or in settings of postwar social disruption (*14*); but no such recent events have occurred in Tasmania. An alternate possibility is introduction of tularemia to Australia by water birds migrating from Southeast Asia that, if infected, have the potential to contaminate surface waters with *F. tularensis* (*15*).

## Conclusions

This case provides evidence of type B tularemia in Australia and should alert physicians and veterinarians working within the region to the possibility of infection with this organism. The transmission of *F. tularensis* to a human host after the bite of an animal native to Australia suggests an ecologic niche for this bacterium in the forests of western Tasmania. Further research is necessary to elucidate the role that ringtail possums, small mammals, ticks, tabanid flies, and mosquitoes prevalent in this unique location might play as natural reservoirs for and vectors of *F. tularensis*.
